# Wounds to Wisdom: Exploring Risk Factors and Outcomes for Surgically Managed Postcesarean Wound Infections

**DOI:** 10.1055/a-2789-1655

**Published:** 2026-02-09

**Authors:** Janice Wong, Emma E. H. Peek, Ysaac Zegeye, Ashley Cummings, Mia Grayson, Caitlin Moran, Sara Bernate Angulo, Carmen Rauh Garrido, Jennifer Okunbor, Rachel Wood, Sarah K. Dotters-Katz

**Affiliations:** 1 Department of Obstetrics & Gynecology, Duke University Medical Center, Durham, North Carolina, United States; 2 Department of Urogynecology and Reconstructive Pelvic Surgery, University of California San Diego, San Diego, California, United States; 3 Duke University School of Medicine, Durham, North Carolina, United States; 4 Division of Maternal Fetal Medicine, Department of Obstetrics and Gynecology, Duke University School of Medicine, Durham, North Carolina, United States

**Keywords:** cesarean section, wound infection, surgical intervention, postcesarean wound infection

## Abstract

**Objective:**

Postcesarean surgical site infections (SSIs) contribute substantially to morbidity and healthcare costs, yet understanding of their management remains limited.

**Study Design:**

Retrospective cohort study of patients delivering via cesarean at a single healthcare system from June 2013 to July 2022 with SSIs within 30 days of delivery. Rates and risk factors for surgical intervention were examined as the primary outcome. Secondary analysis evaluated outcomes in those who required surgical versus conservative management.

**Results:**

Of 533 patients, 69 (12.9%) required surgical management; this population was less likely to have private insurance and more likely to have diabetes than patients managed conservatively. Factors independently associated with surgical intervention included body mass index (BMI) 40 to 49.9, BMI ≥ 50, hypertensive disorders of pregnancy, blood transfusion, general anesthesia, and penicillin allergy. Among 297 patients evaluated, patients requiring surgical intervention( *n* = 69, 23.2%) experienced higher rates of morbidity, including sepsis, acute kidney injury, and fascial dehiscence. Patients requiring surgical intervention had higher rates of inpatient admission, intensive care unit admission, and longer readmissions.

**Conclusion:**

Patients with higher BMI, hypertensive disorders of pregnancy, general anesthesia, blood transfusion, and penicillin allergies may warrant closer monitoring for wound infection. Furthermore, patients requiring surgical intervention for postpartum wound infections had higher morbidity and longer, more complex hospitalizations.

## Introduction

Cesarean delivery, the most commonly performed surgery in the United States and worldwide, remains a significant risk factor for postpartum maternal infection. 1 These infectious complications, ranging from mild wound infections to life-threatening conditions, contribute substantially to maternal morbidity and mortality rates worldwide. Common complications include fever, wound infection, endometritis, urinary tract infection (UTI), and prolonged hospitalization. In severe cases, complications such as pelvic abscess, bacteremia, septic shock, necrotizing fasciitis, and septic pelvic vein thrombophlebitis can occur, posing serious threats to maternal health. 2 3 4

Data from the CDC National Nosocomial Infections Surveillance System, spanning from 1992 to 2004, revealed a pooled mean rate of surgical site infections (SSI) following cesarean section in U.S. hospitals to be 3.15%. Notably, this rate ranged from 2.71% in low-risk patients to 7.53% in high-risk patients. 5 Various factors have been identified as contributing to an increased risk of postcesarean SSI, including emergency cesarean delivery, prolonged duration of ruptured membranes and labor, lower socioeconomic status, fewer prenatal care visits, increased vaginal exams in labor, use of internal fetal monitors, use of general anesthesia, surgical technique and expertise, and presence of UTIs, anemia, hemorrhage, maternal obesity, diabetes, and subcutaneous hematoma. 6 7 8 9 10

While risk factors for wound complications following cesarean delivery have been well-characterized, there remains a gap in understanding the specific risk factors and rates of morbidity associated with postcesarean SSI necessitating surgical intervention, particularly the need for surgical debridement. To date, limited research has further evaluated the incidence, risk factors, and morbidity for indicated surgical intervention among patients with postcesarean wound infections. Therefore, this retrospective study aims to address this gap in knowledge.

The primary objective of this study is to describe the incidence of surgical intervention among patients with postcesarean wound infections. Additionally, secondary objectives include identifying risk factors associated with surgical intervention as well as comparing morbidity outcomes between patients who do and do not require surgical intervention for postcesarean SSI.

## Materials and Methods

This retrospective cohort study includes all patients who underwent cesarean delivery at the Duke Healthcare System between June 21, 2013, and July 1, 2022, with subsequent SSI diagnosed within 30 days postpartum. Patients with wound infections but not delivering at Duke were excluded from the study. Additionally, cases of wound seromas, hematomas, and wound dehiscence without evidence of infection were excluded. For patients with multiple instances of postcesarean SSI, only the first occurrence was considered for analysis. Patients were identified using the Duke Enterprise Data Unified Content Explorer platform and current procedural terminology codes for cesarean delivery, along with International Classification of Diseases 9 and 10 codes associated with SSI within 30 days postcesarean. Demographic data, maternal comorbidities, and intrapartum and postpartum details were abstracted from the medical record. Study data were collected and managed using Research Electronic Data Capture tools hosted at Duke University. 11

In the primary analysis, the primary outcome of interest was the rate of surgical intervention for SSI management. The secondary outcome of interest included risk factors for needing surgical intervention. Surgical intervention was defined as the patient requiring management in the operating room for their wound infection. Radiologic-guided procedures or procedures performed at the patient's bedside were not considered surgical interventions.

Subsequently, a planned secondary analysis was performed to examine morbidity associated with surgical intervention. Due to the inherent higher risk associated with patients presenting for evaluation in the inpatient setting compared with those managed conservatively in the clinic, a separate analysis was conducted specifically among patients evaluated in the hospital setting, including those in obstetric triage, the emergency department, and during the initial hospitalization following cesarean delivery. Patients requiring surgical intervention were again compared with those who did not. Morbidities assessed included need for imaging-guided drain placement, sepsis, acute kidney injury or renal failure, intensive care unit admission, fascial dehiscence, and bowel resection.

Descriptive statistics were used to summarize demographic characteristics and clinical variables. Categorical variables were presented as frequencies and percentages, while continuous variables were reported as medians with interquartile ranges. Comparisons between patients requiring surgical intervention and those managed conservatively were conducted using chi-square tests for categorical variables, and Mann–Whitney U tests for continuous variables, as appropriate. Multivariable logistic regression analysis was performed to identify independent risk factors associated with the need for surgical intervention, with Poisson regression used to estimate risks. All statistical analyses were conducted using Stata, and a two-sided *p* -value of < 0.05 was considered statistically significant. 12

This study was approved by the Institutional Review Board (IRB) at Duke University Hospital (IRB protocol number: Pro00101602) prior to commencement. Informed consent was waived by the IRB due to the retrospective nature of the study and the use of de-identified patient data.

## Results

**Fig. 1:**
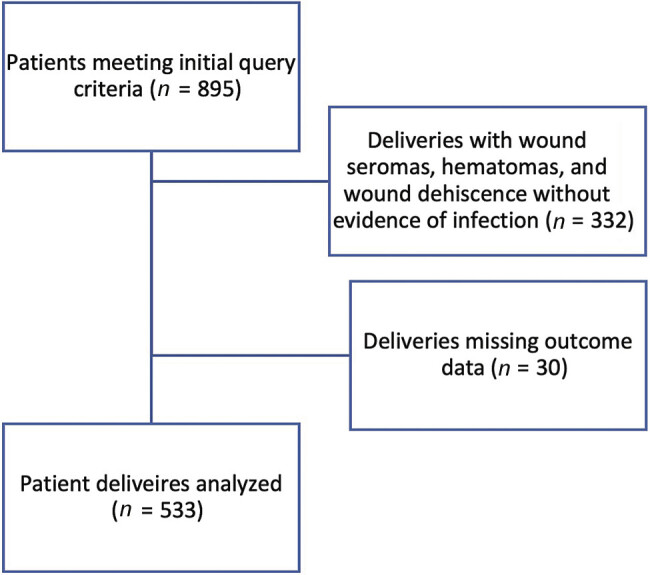
Diagram of analysis population derivation.

Overall, 533 patients were included in the analysis ( Fig. 1 ). Of these, 69 (12.9%) required surgical management for postcesarean wound infections. Notably, patients requiring surgical management were less likely to have private insurance (21.7% vs. 55.4%, *p* < 0.001) and were more likely to have hypertensive disorders of pregnancy (42.0% vs. 21.3%, *p* < 0.001), obesity (91.3% vs. 80.0%, *p* = 0.02), and diabetes (gestational 15.9% vs. 9.7%, pregestational 18.85% vs. 8.0%, *p* = 0.002) compared with those managed conservatively. Other demographic and pregnancy characteristics of the study population are summarized in Table 1 .

**Table 1:** Summary of patient demographic and pregnancy characteristics of the analysis population

	Overall ( *n* = 533)	No surgical management ( *n* = 464)	Surgical management ( *n* = 69)	*p* -Value
	Median [25th–75th Percentile] or %			
Maternal age at delivery	31.0 [27.0, 35.0]	31.0 [27.0, 35.0]	30.0 [25.0, 36.0]	0.54
Race				0.31
White	244 (46.0)	218 (47.2)	26 (37.7)	
Black	180 (33.9)	152 (32.9)	28 (40.6)	
Other	107 (20.2)	92 (19.9)	15 (21.7)	
Ethnicity: Latinx	80 (15.0)	70 (15.1)	10 (14.5)	0.90
Private insurance	272 (51.0)	257 (55.4)	15 (21.7)	<0.001
Hypertensive disorder of pregnancy	128 (24.0)	99 (21.3)	29 (42.0)	<0.001
Chronic hypertension	77 (14.4)	64 (13.8)	13 (18.8)	0.27
Diabetes mellitus				0.002
Gestational diabetes	56 (10.7)	45 (9.7)	11 (15.9)	
Pregestational (1 or 2)	50 (9.4)	37 (8.0)	13 (18.8)	
Maternal mental health disorder	152 (28.5)	136 (29.3)	16 (23.2)	0.29
History of venous thromboembolism	11 (2.1)	6 (1.3)	5 (7.2)	0.001
HIV	2 (0.4)	1 (0.2)	1 (1.4)	0.12
Hepatitis C	4 (0.8)	3 (0.6)	1 (1.4)	0.47
Systemic lupus erythematosus	8 (1.5)	6 (1.3)	2 (2.9)	0.31
Asthma	79 (14.8)	70 (15.1)	9 (13.0)	0.66
Prior C-section	235 (44.1)	216 (46.6)	19 (27.5)	0.003
Substance use during pregnancy	86 (16.3)	70 (15.2)	16 (23.5)	0.08
Multiparous	308 (81.3)	279 (82.8)	29 (69.0)	0.03
Multiple gestation	31 (5.8)	29 (6.2)	2 (2.9)	0.27
BMI at admission	37.5 [32.2, 45.2]	36.5 [31.7, 43.2]	45.4 [38.3, 54.4]	<0.001
< 30	99 (18.6)	93 (20.0)	6 (8.7)	
30–39.9	221 (41.5)	205 (44.2)	16 (23.2)	
40–49.9	128 (24.0)	106 (22.8)	22 (31.9)	
> 50	85 (15.9)	60 (12.9)	25 (36.2)	

Abbreviations: BMI, body mass index; HIV, human immunodeficiency virus.

Patients who required surgical intervention for postcesarean SSI were more likely to have group B strep positivity (35.5% vs. 23.4%, *p* = 0.04) and to have labored prior to c-section (60.9% vs. 46.0%, *p* = 0.02) compared with those managed conservatively. Additionally, the use of general anesthesia at the time of cesarean was associated with a higher rate of surgical intervention of SSI compared with conservative management (15.9% vs. 5.2%, *p* < 0.001). Operative time, skin incision type, blood loss, presence of intraamniotic infection, skin closure with staples, and performance of cesarean hysterectomy were not significantly different between groups ( Table 2 ).

**Table 2:** Summary of admission, delivery, and postpartum characteristics

	Overall ( *n* = 533)	No surgical intervention ( *n* = 464)	Surgical intervention ( *n* = 69)	*p* -Value
	Median [25th–75th Percentile] or %			
White cell count at admission	9.9 [8.3, 11.8]	10.0 [8.3, 11.8]	9.8 [8.3, 11.8]	0.79
Hemoglobin (Hb) at admission	11.7 [10.9, 12.5]	11.7 [10.9, 12.5]	11.9 [10.7, 12.6]	0.89
Anemia on admission (Hb < 11)	137 (25.7)	115 (24.8)	22 (31.9)	0.21
Group B strep positive	116 (25.1)	94 (23.4)	22 (35.5)	0.04
Gestational age at delivery	38.7 [37.1, 39.4]	38.9 [37.1, 39.6]	37.9 [37.0, 39.3]	0.17
Labored	255 (47.9)	213 (46.0)	42 (60.9)	0.02
Intraamniotic infection present	59 (11.1)	50 (10.8)	9 (13.2)	0.55
Fetal scalp electrode used	37 (6.9)	17 (3.7)	20 (29.0)	<0.001
Intrauterine pressure catheter used	57 (10.7)	33 (7.1)	24 (34.8)	<0.001
General anesthesia used	35 (6.6)	24 (5.2)	11 (15.9)	<0.001
Operative time (incision to end of procedure in minutes)	69.0 [57.0, 87.0]	69.0 [56.0, 85.0]	75.0 [60.0, 93.0]	0.08
Estimated blood loss greater than 1,000 mL	107 (20.1)	89 (19.2)	18 (26.1)	0.18
Estimated blood loss	700.0 [500.0, 895.0]	700.0 [500.0, 850.0]	800.0 [500.0, 1,000.0]	0.28
Midline vertical skin incision	35 (6.6)	29 (6.2)	6 (8.7)	0.44
Supraumbilical skin incision	5 (0.9)	3 (0.6)	2 (2.9)	0.07
Cesarean hysterectomy performed	9 (2.4)	8 (2.4)	1 (1.9)	0.79
Received azithromycin	126 (23.6)	105 (22.6)	21 (30.4)	0.15
Received cefazolin	407 (76.4)	362 (78.0)	45 (65.2)	0.02
Penicillin allergy	116 (21.8)	95 (20.5)	21 (30.4)	0.06
Received a blood transfusion	24 (4.5)	17 (3.7)	7 (10.1)	0.02
Vaginal preparation used	289 (54.2)	248 (53.4)	41 (59.4)	0.35
Skin closed with staples	33 (6.2)	26 (5.6)	7 (10.1)	0.14
Received postpartum antibiotics	55 (10.5)	40 (8.7)	14 (21.2)	0.002
Discharged with prophylactic low molecular weight heparin	167 (31.4)	138 (29.7)	29 (42.6)	0.03

Patients with a body mass index (BMI) of 40 to 49.9 (iRR: 3.81, 95% CI: 1.32–11.1) and BMI greater than 50 (iRR: 5.63, 95% CI: 1.91–16.5) had a significantly higher risk of surgical intervention. Other factors independently associated with surgical intervention included hypertensive disorders of pregnancy (iRR: 2.05, 95% CI: 1.15–3.63), penicillin allergy (iRR: 2.14, 95% CI: 1.15–3.97), blood transfusion (iRR: 3.71, 95% CI: 1.25–11.1), and general anesthesia use (iRR: 3.33, 95% CI: 1.42–7.79). Adjusted risk ratios for labor and postpartum antibiotic use were not significant ( Table 3 ).

**Table 3:** Poisson regression associations between prespecified factors and the surgical intervention outcome

	Relative risk (95% confidence interval)
BMI a	
30–39.9	1.24 (0.42–3.66)
40–49.9	3.81 (1.32–11.1)
50 and greater	5.63 (1.91–16.5)
Penicillin allergy	2.14 (1.15–3.97)
Preexisting diabetes	1.55 (1.07–2.25)
Labor	1.55 (0.87–2.77)
Hypertensive disorder of pregnancy	2.05 (1.15–3.63)
General anesthesia	3.33 (1.42–7.79)
Intraamniotic infection/postpartum antibiotics	1.86 (0.86–4.00)
Blood transfusion	3.71 (1.25–11.1)

Abbreviation: BMI, body mass index.

a Referent group is BMI < 30.

**Fig. 2:**
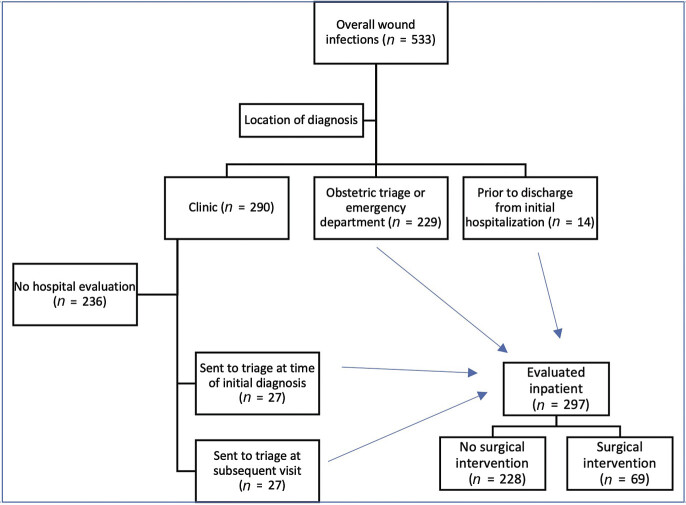
Schematic overview of the location of initial diagnosis and evaluation of surgical site infection.

Of all patients with wound infections, 297 (55.7%) were evaluated in hospital settings, including obstetric triage, the emergency department, and inpatient ( Fig. 2 ). Patients in this group requiring surgical intervention ( *n* = 69, 23.2%) experienced higher rates of morbidity compared with those managed conservatively. This included a higher incidence of sepsis (8.7% vs. 1.3%, *p* = 0.002), acute kidney injury or renal failure (7.2% vs. 1.8%, *p* = 0.02), and fascial dehiscence (29.0% vs. 0.0%, *p* < 0.001). Further, patients requiring surgical intervention had a higher rate of inpatient admission (97.1% vs. 32.0%, *p* < 0.001), a higher rate of intensive care unit admission (5.8% vs. 1.3%, *p* = 0.03), and a significantly longer length of readmission (5.0 days vs. 3.0 days, *p* = 0.004; Table 4 ).

**Table 4:** Morbidity outcomes for patients evaluated inpatient requiring surgical intervention versus no surgical intervention

	Patients evaluated inpatient; *n* = 297 (%)	No surgical intervention; *n* = 228 (%)	Surgical intervention; *n* = 69 (%)	*p* -Value
Imaging-guided drain placement	17 (5.7)	14 (8.1)	3 (5.6)	0.53
Sepsis	9 (3.0)	3 (1.3)	6 (8.7)	0.002
Acute kidney injury or renal failure	9 (3.0)	4 (1.8)	5 (7.2)	0.02
Intensive care unit admission	7 (2.4)	3 (1.3)	4 (5.8)	0.03
Venous thromboembolism	7 (2.4)	4 (2.1)	2 (3.0)	0.66
Fascial dehiscence	20 (6.7)	0 (0.0)	20 (29.0)	<0.001
Bowel resection	1 (0.03)	0 (0.0)	1 (1.4)	0.07
Inpatient admission	140 (47.1)	71 (32.0)	67 (97.1)	<0.001
Length of readmission (d)	3.5 [2.0, 5.0]	3.0 [1.0, 5.0]	5.0 [3.0, 6.0]	0.004
Negative pressure wound therapy used	97 (32.7)	33 (14.8)	54 (79.4)	<0.001

## Discussion

At our institution, the rate of surgical intervention for SSI was 12.9% (or approximately one in two patients). The principal findings of this study reveal significant associations between various demographic, pregnancy, and clinical factors and the need for surgical intervention in patients with postcesarean wound infections. In particular, patients with higher BMI, hypertensive disorders of pregnancy, penicillin allergies, and those who underwent general anesthesia or required blood transfusions were more likely to require surgical management. Additionally, patients requiring surgical intervention experienced higher rates of morbidity, including sepsis, acute kidney injury, and fascial dehiscence, compared with those managed conservatively.

Our findings align with previous studies indicating an increased risk of postcesarean wound complications in patients with higher BMI and hypertensive disorders of pregnancy. 1 6 7 8 Obesity is a known risk factor for wound complications, and in this population, limited available data support primary or secondary closure of wounds over healing by secondary intention as well as the use of vacuum-assisted wound closure devices. 13 However, the available evidence supporting these interventions remains limited, emphasizing the need for well-designed randomized controlled trials to establish their efficacy. Hypertension is also a known risk factor for SSI, and while the exact mechanism is unclear, some studies postulate that peripheral vascular disease and associated prolonged bleeding may cause delayed wound healing. 14 15

Our study also found that patients who received blood transfusions were at increased risk of postcesarean wound complications. As admission hemoglobin and rates of anemia were not different between our study groups, we suspect that this finding reflects estimated blood loss during surgery, which may be a marker for duration and complexity of the surgery, or need for antibiotic re-dosing. There is some data to suggest that preoperative anemia is associated with postcesarean wound infections, although more research is needed on this topic. 16

The association between general anesthesia and SSIs has been previously recognized in existing literature. Various studies have proposed potential mechanisms that may mitigate infection risk with neuraxial anesthesia, such as an attenuated inflammatory response to surgery, improved tissue oxygenation through the vasodilation induced by neuraxial techniques, and enhanced analgesia postoperatively with decreased pain-associated autonomic response and vasoconstriction. 17 Additionally, general anesthesia is typically administered in the setting of emergent cesarean deliveries, accompanied by a “splash” povidone-iodine skin preparation. Despite this, a recent retrospective cohort study evaluating prophylactic postoperative antibiotics in emergent cesarean deliveries did not demonstrate lower rates of postpartum infections or wound complications. 10 However, the association between general anesthesia and surgical intervention of postcesarean wound infections remains relatively unexplored, indicating the need for more robust studies in this area.

Furthermore, our study revealed a new association between penicillin allergy and the surgical management of postcesarean wound infections. Other studies have demonstrated that a reported penicillin allergy is associated with an increase in healthcare-associated infections and SSIs. 18 19 Implementation of an allergy history-guided algorithm in obstetrical patients with reported β-lactam allergies has been shown to result in an increase in perioperative cefazolin prophylaxis. 20 This novel finding underscores the importance of considering individual characteristics and allergy profiles in clinical decision-making regarding wound management strategies.

Finally, our study revealed significant morbidity outcomes among patients undergoing surgical management of postcesarean SSI, including higher rates of sepsis, acute kidney injury or renal failure, intensive care unit admission, and fascial dehiscence. These patients also experienced an increase in inpatient admissions and prolonged lengths of admission compared with those managed conservatively. These results underscore the substantial burden of morbidity associated with surgical intervention in this patient population and emphasize the need for further investigation into optimal management strategies to mitigate these adverse outcomes.

The results of this study underscore the importance of identifying high-risk patients who may benefit from early identification and early surgical intervention for postcesarean wound infections. Clinicians should be vigilant in monitoring patients with higher BMI and hypertensive disorders for signs of wound complications and consider the potential need for surgical management. Additionally, the associations between general anesthesia and penicillin allergy with surgical intervention warrant further investigation to optimize perioperative care strategies and mitigate postoperative morbidity in this population.

While our study provides insights into the factors influencing the need for surgical intervention in patients with postcesarean wound infections, additional research is needed to validate these findings in diverse patient populations and healthcare settings. In particular, we recognize that our study represents the findings from a single site of 533 patients. As the primary purpose of our study was to identify risk factors that might provide insight into how to identify patients at greatest risk for SSI postcesarean, we did not assess the effects of the many contemporary practice changes that took place over the 11-year study period. Large, multicenter studies are needed to assess how management changes in antibiotics for cesarean wound infections have affected this patient population. Future studies should also focus on exploring novel interventions to prevent and manage postcesarean wound complications effectively.

A strength of this study is its comprehensive analysis of a large cohort of patients from a single healthcare system caring for a diverse patient population with a large referral base over 10 years, allowing for detailed examination of demographic and clinical factors associated with surgical intervention for postcesarean wound infections. However, the retrospective nature of the study may introduce inherent biases and limitations, such as incomplete documentation and potential confounding variables not accounted for in the analysis. Additionally, the generalizability of our findings may be limited to similar healthcare settings and patient populations.

In conclusion, our study provides important insights into the risk factors and clinical outcomes associated with surgical intervention for postcesarean wound infections. Anecdotally, patients who require repeat surgery for postcesarean infections tend to have higher morbidity in contrast to those who are successfully managed conservatively. However, this has yet to be published, and our data emphasize the need for targeted interventions and increased surveillance in patients at higher risk to reduce complications in this patient population. Further research is warranted to validate our findings and inform evidence-based strategies for the management of postcesarean wound infections in clinical practice.
